# Training needs and perceptions on teaching in primary care from doctors who teach in primary care in Peru

**DOI:** 10.15694/mep.2021.000038.1

**Published:** 2021-02-05

**Authors:** Maria Sofia Cuba-Fuentes, Angela Ortigoza, Alessandra Pirazzoli, Elizabeth Grant, Philippa Moore

**Affiliations:** 1 Universidad Peruana Cayetano Heredia (UPCH); 2 Pontificia Universidad Catolica de Chile; 3 Universidad Alberto Hurtado; 4 University of Edinburgh

**Keywords:** Medical education, Primary Health Care, Family Medicine, Peru

## Abstract

This article was migrated. The article was marked as recommended.

Introduction

Medical training should include a strong emphasis on primary health care. There is a need for primary care teaching centres and teachers who can provide excellent instruction in primary health care (PHC).

Objectives

This investigation explores the characteristics of the doctors who teach in PHC in Peru, their educational needs and their perceptions of their teaching.

Method

Using a mixed method approach we ran an online questionnaire on the educational needs and focus groups which explored the challenges, problems and skills required for teaching in PHC.

Results

66 teachers from 10 regions answered the questionnaire: 59 (89.4%) were family doctors; 76,7% had a formal university contract; they dedicated an average of 12.9 hours/week to teaching and 9 (13,6%) had had some training in teaching during the last 5 years. In the focus groups they showed interest in developing their teaching skills and 4 dimensions were defined: willingness to teach; teaching family medicine; teacher-student relationships; the organization of the teaching.

Conclusion

The PHC teachers in Peru have great interest in teaching and a need for training in teaching skills.

## Introduction

Health systems require well-trained primary health care teams in order to take care of most of the health problems of the population they serve ( OMS, 2008).

In Latin America, there are insufficient human resources in primary health care (PHC) both in number and in training ( Giraldo Osorio and Vélez Álvarez, 2013). A strategy implemented to strengthen human resources in PHC has been to increase both undergraduate and post-graduate training in outpatient community settings.

The Pan American Health Organization (PAHO) proposed as a goal for 2015 that 80% of health science schools should reorient their training towards PHC, including the health needs of communities and a strong emphasis on teamwork and interprofessional training ( Organización Panamericana de la Salud/Organización Mundial de la Salud (OPS/OMS), 2007). Achieving this goal requires clinical scenarios in PHC and teachers working in those scenarios, with excellent teaching skills interacting with undergraduate and post graduate students.

In Latin America, some training programs in Family and Community Medicine lack elements to achieve the standardized competencies defined worldwide ( Ruilova *et al.*, 2016). In Peru, there are no university departments, of Family and Community Medicine, despite the fact that there are approximately 150 family medicine residents in training in 20 residency programs throughout the country ( Romero-Albino and Cuba-Fuentes, 2019). There are also some experiences of successful undergraduate curricular activities carried out in PHC in Peru ( Champin and Risco de Domínguez, 2014) and these innovations in PHC teaching-care integration in Peru are acceptable and satisfactory for both users ( Saco-Méndez and Zavala-Sarrio, 2017) and students ( Santos *et al.*, 2019). However, as far as we know, there is no information published about the professionals who work and teach in PHC in Peru. This research explores the characteristics of the doctors who teaching in PHC their training needs and their perceptions of PHC teaching.

## Methods

We used a mixed method approach to collect the data; first an online survey was conducted on the needs of doctors who work and teach in PHC in Peru (see Supplementary File 1). The survey was created based on the domains of teaching competence defined by Sherbino, Frank and Snell (2014) and was validated with a panel of PHC expert physicians ( Moore *et al.*, 2020). The survey included questions on sociodemographic variables, the importance of 10 competencies for teaching, and the form and feasibility of conducting training in teaching skills for professionals working in PHC. Surveys were sent via email between October and November 2018 and follow-up reminders by email 1, 2 and 4 weeks after the first invitation to participate.

The survey was sent to a convenience sample of doctors whose emails were obtained by the ASPEFAM network (Peruvian Association of Faculties of Medicine), and the official social networks of SOPEMFYC (Peruvian Society of Family and Community Medicine). To be included in the study the participants had to be doctors teaching undergraduate or postgraduate students in PHC and to accept the online consent. The responses to the survey were analysed using descriptive statistics, preserving the anonymity of the participants.

For the focus groups, participants in a course to improve teaching skills held during May 2018 were invited to participate in a focus group session of 1.5 hours. prior to the course. 30 of 66 course participants gave their consent to participate, with 10 participants each focus group. The questions guiding the discussion addressed the approach to teaching, teaching methods, problems faced in the teacher-student relationship and the knowledge, attitudes and skills the participants felt they required for their teaching role.

Each focus group was audio-recorded, and the recording was transcribed verbatim, maintaining anonymity. Content analysis was used to analyse the discussion.

This study had the authorization of the ethics committees of the Pontificia Universidad Católica de Chile and the Universidad Peruana Cayetano Heredia (CIE-UPCH). All participants of the survey and focus groups signed an informed consent.

## Results/Analysis

### Survey

**Figure 1.**
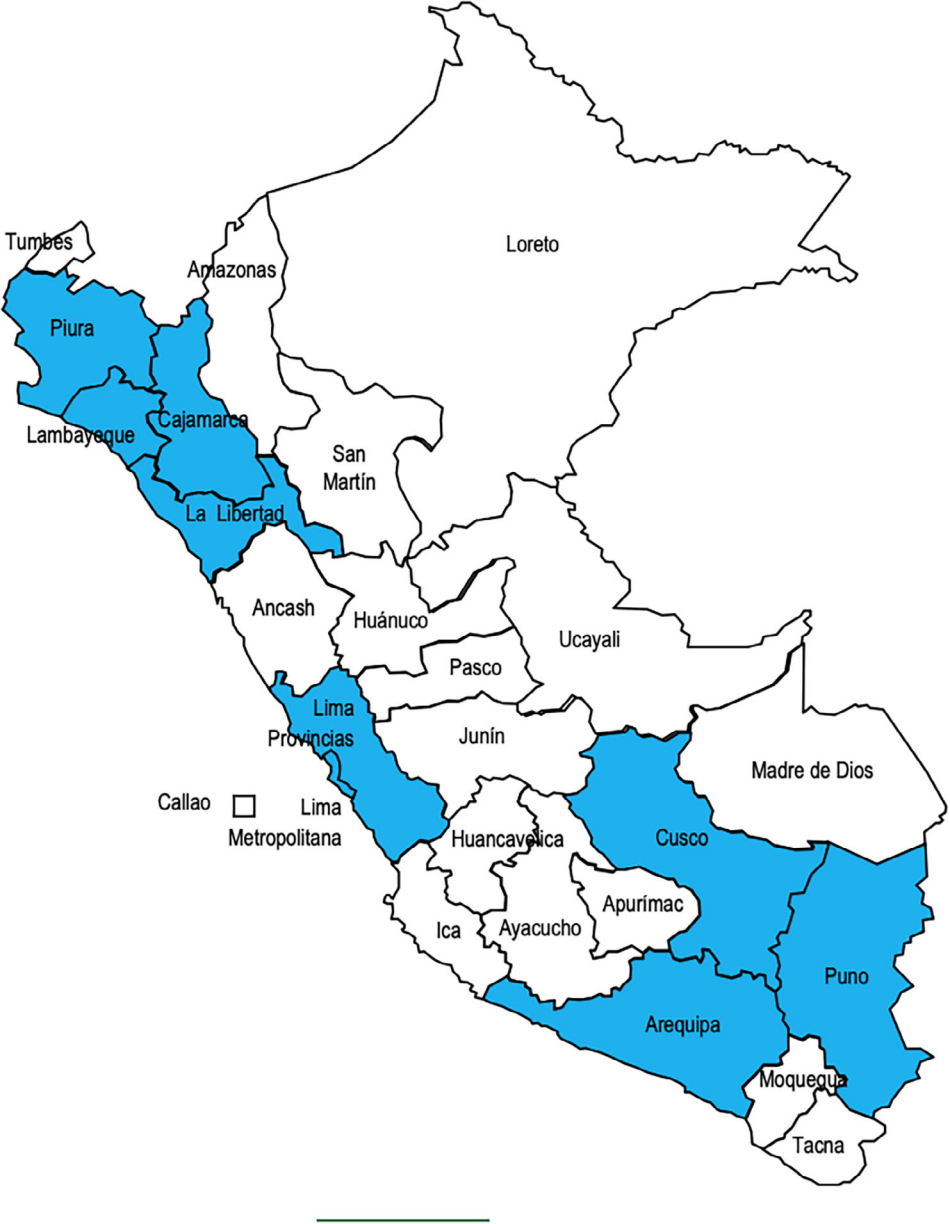
Regions in Peru where the survey respondents are teaching in PHC

Of the ninety-eight PHC teachers who were sent the survey, 66 answered the survey (response rate 67.35%). The professionals who responded came from 10 regions of the country ( Figure 1).

89.4% (59) were family doctors; 51.5% (34) were women, 44% (29) were between 31 and 35 years of age, and 53% (35) were 36 years of age or older.

84.8% (56) performed teaching linked to a university. Of these, 76.7% did so with a formal contract. 78.8% (42) worked as clinical doctors in a PHC facility at the time of the survey. The average weekly dedication to teaching in any setting was 12.9 hours (range 1-40 hours). The time devoted to teaching in the PHC facilities was 9.61 hours per week (range of 1-20 hours). 40 teachers (60.6%) taught more than one target group, including undergraduate and graduate medicine and other settings. While those who taught only one target group (26), more than half (14 respondents) taught only in a Family Medicine residency programme. 84.8% (56) stated that their students accompanied them in their daily clinical practice as part of the learning process. Only 13.6% (9 respondents) had had some training in teaching skills in the last 5 years.

The respondents’ perceptions of the importance of teaching competencies for professionals who teach in PHC are shown in Table 1.

**Table 1.** Respondent perception of the importance of teaching skills training for PHC teachers in Peru

Teaching Competencies	Agree		Disagree		Total	
Leadership	63	(95.5%)	3	(4.5%)	66	(100%)
Communication skills	62	(93.9%)	4	(6.1%)	66	(100%)
Giving Feedback	62	(95.4%)	3	(4.6%)	65	(100%)
Observation of the consultation	61	(95.3%)	3	(4.7%)	64	(100%)
Teaching procedures	61	(93.8%)	4	(6.2%)	65	(100%)
Running classes or seminars	60	(93.8%)	4	(6.3%)	64	(100%)
Evaluation	60	(93.8%)	4	(6.3%)	64	(100%)
Curriculum	58	(90.6%)	6	(9.4%)	64	(100%)
Preparing educational materials	20	(31.7%)	43	(68.3%)	63	(100%)

87% of the professionals agreed or strongly agreed that all teaching competencies were important, highlighting the importance of leadership (95.5%), delivery of feedback (95.4%) and observation of clinical care (95.3%).

When asked about the preferred methods for training teaching skills, the blended format (B-learning) was the preferred type (84.8%) ( Table 2).

**Table 2.** Preferred methods for training in teaching skills

Teaching Method	N°	%
Blended learning (B-learning)	56	84.8%
Face to face	8	12.1%
Virtual (E-learning):	2	3.0%

### Focus groups

30 teachers participated in the focus groups. Although all showed an excellent disposition towards teaching and interest in developing skills to improve their teaching, they described their teaching as based on intuition and common sense.

Four dimensions of emerging issues on teaching in PHC arose from the focus groups: a willingness to teach; teaching in Family Medicine; teacher-student relationship; organization of rotations ( Table 3).

**Table 3.** The four dimensions arising from the focus groups with illustrative citations

Attitude towards teaching
*“We are all really ready to learn we are all ready for it and we all want to improve, for the good of the residents and for us too!.”* Person 3, Group 3
*“Well, I would also like to get some skills, some kind of strategy to improve as a teacher so that I can reach out to the student, manage my time better maybe, so maybe I can get some teaching strategies, I’ll put the initiative, the will and the enthusiasm, but I know that like all processes it’s good to learn new methods.”* Person 1, Group 3
*“I think that as teachers we all need to learn new skills and know what to do in those cases; for everything else I think you could have written guidelines. You can learn to do feedback or role-playing but I think what I would like to learn is if there are any skills that would help me with difficult patients or residents.”* Person 9, Group 1
*“How can I tell you - I haven’t had any formal training as a teacher, I haven’t specialized in education as lots of other teachers have. So, my teaching comes from my experience.”* Person 4, Group 4

**Teaching in the Family medicine program**
*“One thing is the academic part where a part of what they learn is clinical and another part is the Family Medicine rotation where they apply what they have learnt and it’s just the family medicine rotations where they spend less time. So, I think that the curriculum should be changed so they have more time in family medicine, so that my residents, for example would return to spend more time with me.”* Person 5, Group 3 *“Both Family Medicine residents and those who study in the hospital-centred model, most of them have a biomedical way of thinking towards Family Medicine. The ones who are already on their rotation don´t really know what Family Medicine is and those who are starting are even worse - they don’t know what they are getting involved in, they know they are starting a specialist residency program called Family Medicine, but they don´t know what that is.*” Person 8, Group 1
*“In our case, the teaching sites offered by the university have always been only hospital-based. There are no primary care teaching sites, they are all hospital sites. So, we need to pull them outside a bit, give then a bit of space, to return to that space, so that’s what we are working on - so that there really is primary care.”* Person 1, Group 1

**Student-teacher relationship**
*“So, as they go acquiring skills about the system, the medical interview, they eventually can take charge of seeing patients - obviously with us there, by their side.*” Person 2, Group 2
*“when we are with the patient, sometimes I think they might make a mistake, and so I intervene, so in a way i think I am very maternal . . .”* Person 5, Group 1
*“When I feel that they have offended a patient or have been too harsh - because of the patient and their reactions - then I´ll try to intervene to be a buffer because I believe that that’s part of working with patients of different cultures and beliefs . .”* Person 6, Group 3

**The organization of the rotations**
*“... .We have tried to unify criteria, because the university has its own style . . let’s say we have tried to unify criteria; with 5 other family doctors we have tried to create a timetable of activities so that the resident is with us in our medical interviews but also they have some time to do their presentations of clinical cases or clinical topics of their choice.”* Person 5, Group 1
*“in practice they are with me in my office, observing how I see patients, they can also get involved and participate, so that’s the best way, because you can’t prepare - “we are going to see such and such a case today”. We simply sit down early and see the patients that arrive, and depending on who arrives we talk and develop ideas on the spot and afterwards depending on what we have seen, we develop topics that the residents themselves choose.”* Person 5, Group 2

They described the need to provide a curricular structure to the Family Medicine specialty and to generate a triangulation between the Family Medicine curriculum and the needs and structure of the health system. A national Family Medicine curriculum with clear competencies would serve as a guide so that training in different centres can be organized under common standards.

Some teachers stated that they have a “paternalistic” attitude (in their own words,) with their residents, maintaining close observation and intervening to avoid mistakes in their contact with patients.

Each tutor generated their own teaching structure. They used their creativity to generate teaching moments at the same time as seeing patients.

## Discussion

This is the first study in Peru that describes the situation of physicians who teach in PHC. It was possible to collect information from teachers linked to 16 Universities from 10 different cities. The teachers carried out various activities that ranged from teaching undergraduate students, residents, training health teams and peers in health facilities, on a very part-time basis (average 9.6 hours per week).

76.4% of the teachers did not have any training in teaching. There is growing evidence that PHC teachers would benefit from systematic training in teaching knowledge and skills for adult education ( Bloom, 2005) and when they receive this training, they not only improve their competence as teachers but also improve their clinical skills and skills. job satisfaction ( Rubak *et al.*, 2008). Teaching in PHC presents specific challenges, which differ from teaching in the hospital setting. Therefore, the development of competencies required for teaching in PHC in Peru should take into account the environment in which the teaching is carried out, and the needs of the professionals who teach there ( Vella, 2007).

Although there is an excellent disposition towards teaching in PHC, teachers in Peru carry out their teaching using intuition and common sense rather than any educational theory or based on formal training. They identify the competences that are important for teaching in the survey, but in the focus groups they do not name key aspects of teaching with conceptual terms. It is likely that the absence of any structural organization of Family Medicine in the universities leads to a lack of standardized or structured teaching ( Cuba Fuentes and Romero Albino, 2016). The need to develop a clear curriculum for Family Medicine training programs in Peru is urgent if we want to ensure quality training for PHC.

Teachers recognize a paternalistic relationship with their residents, with a lot of support in their clinical care. Taking into account the changes in the teaching-learning process in adult education with the importance of the facilitator role of teachers, this recognition of their current relationship style is a first step to change to their new role as facilitator in the learning process.

This study gave us an insight not only into teaching activities of PHC teachers but also into their clinical activities. One in five teachers who teach in PHC did not practice there; probably some PHC teachers work in the hospital or management field and only go to PHC establishments to carry out their teaching. Physician training programs for PHC have been shown to be successful when their teachers are family physicians who serve as clinicians in PHC.

34.8% of the teachers did not have a formal link with a university despite the fact that in Peru the medical residency has a university link. For years, family medicine training programs have been dependent on doctors working in the community and in PHC teaching voluntarily ( Anthony *et al.*, 2014). Initially, emphasis was placed on the benefits that exist when a PHC doctor teaches: they enjoy their work more, they are encouraged to update their medical knowledge, and their patients value them more for their teaching role ( Grayson *et al.*, 1998). However, more recent studies regarding the difficulty of recruiting teachers in PHC show that little / no remuneration is an important issue for PHC physicians ( Drowos *et al.*, 2017; Graziano *et al.*, 2018).

In countries where postgraduate training is strongly linked to universities, having a family medicine department can be a determining factor in the success of family medicine training programs ( Pantoja C, 2003; Ponzo, 2020). With the absence of Family Medicine departments, it is unlikely that Peru will be able to have PHC teachers who have enough time that develop greater academic and research improvements in PHC ( Romero-Albino and Cuba-Fuentes, 2019). Family medicine departments can lead to benefits such as formal contracts for PHC teachers, protected time for teaching, generating an appropriate CV for PHC, training in teacher skills, and generating research.

### Limitations of this study

Although we consider that the teachers included in the survey are representative of all those who teach in PHC in Peru, it will be important to carry out a broader study, covering most regions. The focus group participants had chosen to take a short course in teaching skills, so it is possible that they may not represent the point of view of all physicians who teach at PHC.

## Conclusion

This study is a first step in understanding those who teach in PHC in Peru and what are their needs in their training in teaching skills. The enthusiasm for their teaching and their interest in training in this area will be pillars that will help the development of excellent teaching in PHC in Peru. Strengthening teaching in PHC is imperative for the universities of Peru, and the creation of academic departments in Family Medicine will allow this process to be more effective.

## Take Home Messages

• Teaching in PHC presents specific challenges, the development of competencies required for teaching in PHC in Peru should take into account the environment in which the teaching is carried out.
• PHC teachers are enthusiastic about their teaching.
• 76.4% of the PHC teachers in Peru did not have any training in teaching.
• Strengthening teaching in PHC is imperative for the universities of Peru.
• There is an urgent need for the development of a clear curriculum for Family Medicine training programs in Peru.

## Notes On Contributors

**Dr Maria Sofia Cuba-Fuentes** is a Peruvian family physician and currently Assistant Professor of Family Medicine and director of the Family Medicine Programme at the Universidad Peruana Cayetano Heredia (UPCH), School of Medicine. Her special interest is the development of Primary health Care and Family medicine in Peru. ORCID ID: https://orcid.org/0000-0001-7394-7092

**Dr Angela Ortigoza** is a family physician trained and currently working as a junior academic at the Department of Family Medicine, P. Universidad Católica de Chile. She works in primary care in Santiago. Her special interest is in teaching methods for primary health care teachers. ORCID ID: https://orcid.org/0000-0003-1048-4216

**Alessandra Pirazzoli** is a sociologist currently working as a coordinator in the curricular innovation project at Alberto Hurtado University, Santiago, Chile. She has a Masters in Sociological Research, University of Barcelona and has experience in research and teaching. ORCID ID: https://orcid.org/0000-0002-8514-9642

**Dr Elizabeth Grant** is a British GP and currently working as Assistant Principal Global Health and Director Global Health Academy at the University of Edinburgh, She was one of the directors of the Newton project that lead to courses on teaching in primary health care in Peru. ORCID ID: https://orcid.org/0000-0001-7248-7792

**Dr Philippa Moore**, MRCGP, is a British GP who has lived and worked in Primary Care in Chile for over 30 years and is currently Assistant Professor of Family Medicine at P. Universidad Católica de Chile. She has a special interest in adult education, experiential methods and communication skills teaching. ORCID ID: https://orcid.org/0000-0002-3578-7240
