# Supplementary material for: Training needs and perceptions on teaching in primary care from doctors who teach in primary care in Peru
Source: MedEdPublish (2016). 2021 Feb 5;10:38. [Version 1] doi: 10.15694/mep.2021.000038.1 (PMC10939610; doi:10.15694/mep.2021.000038.1)
Supplement: Peer review history

# Reviewer report: Reviewer response for version 1

**DOI:** 10.21956/mep.18856.r26886

**Published:** 2021-02-09

**Authors:** Johnny Lyon Maris

**Affiliations:** 1 Southampton GP Education Unit

This review has been migrated. The reviewer awarded 4 stars out of 5 Thank you for submitting this very clear position of the perceived needs of those teaching in primary care in Peru. As a Family Physician I am delighted there is an established residency programme and that PHC is included in the undergraduate curriculum. For me there are 2 key issues of note: the first is that over 3/4 of those teaching have had no training in this, and that 1 in 5 do not practise in primary care clinically.Using virtual platforms some basic skills of teaching (e.g. giving feedback, adult learning, presentation skills) would provide some development and calibration opportunities without the need to travel.It would be excellent to see a skills workshop intervention with evaluation and impact on the learners as a next step.I would like to know about the feedback for the teachers who do not clinically practise in primary care, does this make a difference? The answer may impact on future recruitment.

Reviewer Expertise:

NA

No decision status is available

# Reviewer report: Reviewer response for version 1

**DOI:** 10.21956/mep.18856.r26887

**Published:** 2021-02-08

**Authors:** Leila Niemi-Murola

**Affiliations:** 1 University of Helsinki

This review has been migrated. The reviewer awarded 3 stars out of 5 This is an interesting article focusing on training needs and perceptions on teaching in primary care. The insufficient resourcing in primary care seems to be a global phenomenon. The reader would be interested about the situation in Peru – is education the only problem or do they also have a poor brand, in the working conditions, salaries or the location of the centers? The survey included questions about perceived learning needs of domains of teaching competence. However, Table 1 reports the perceived importance of these skills. The survey respondents worked in ten regions – why were these regions chosen? Do they represent the typical setting in Peru? Most teachers were linked with a university. Do the universities have formal contracts with the teachers and if they do, are there any requirements to be qualified as a teacher? Is there any formal pedagogical education for university teachers in Peru?This is an interesting article raising lots of further questions for a reader not familiar with the circumstances in Peru.

Reviewer Expertise:

NA

No decision status is available
